# Retrospective Evaluation of Cryptorchid Sidedness at Colorado State University Between 1984 and 2014 and Oakridge Equine Hospital Between 2008 and 2023

**DOI:** 10.3390/vetsci12090796

**Published:** 2025-08-23

**Authors:** Hannah Fain, Dean A. Hendrickson, Matthew T. Buesing, Gregg Griffenhagen

**Affiliations:** Department of Clinical Sciences, College of Veterinary Medicine and Biomedical Sciences, Colorado State University, Fort Collins, CO 80523, USA; hannah.fain@gmail.com (H.F.); mbuesingdvm@gmail.com (M.T.B.); gregg.griffenhagen@colostate.edu (G.G.)

**Keywords:** cryptorchid, equine, laparoscopy, Quarter Horse, Thoroughbred, warm blood

## Abstract

Cryptorchidism is a common congenital disorder in male horses characterized by the failure of one or both testes to descend into the scrotum. This developmental anomaly has important clinical, surgical, and economic implications, particularly in breeding animals. This retrospective study investigates the prevalence and laterality of cryptorchidism in a large equine population presented to two veterinary referral hospitals—Colorado State University Veterinary Teaching Hospital (CSU VTH, 1984–2014) and Oakridge Equine Hospital (OEH, 2008–2023). Medical records were reviewed to identify affected horses, and data on breed, age, and laterality of retained testes were collected. Anatomical location of retention (inguinal vs. abdominal) was excluded due to inconsistent documentation across the study period. A total of 777 horses met the inclusion criteria, with Quarter Horses comprising the majority of clinical cases. Quarter Horses exhibited a strong predisposition for left-sided testicular retention, whereas Thoroughbreds and Arabians more commonly presented with right-sided retention. These breed-specific trends in laterality suggest possible developmental factors influencing testicular descent. Understanding these patterns can aid in clinical diagnosis, improve surgical planning, and contribute to evidence-based breeding recommendations aimed at reducing the incidence of cryptorchidism in equine populations.

## Simple Summary

This retrospective study examined cryptorchidism in 777 horses presented to two veterinary hospitals over nearly 40 years. Quarter Horses were most commonly affected and showed a strong tendency for left-sided testicular retention, while Thoroughbreds and Arabians more often retained the right testis. These breed-specific patterns may reflect underlying genetic or developmental differences. Recognizing these trends can help veterinarians diagnose and treat cryptorchidism more effectively and inform breeding decisions to reduce its incidence in horses.

## 1. Introduction

Cryptorchidism is a congenital condition characterized by the failure of one or both testes to descend into the scrotum. It is one of the most frequently diagnosed developmental anomalies in the equine male reproductive tract and poses challenges for diagnosis, treatment, and breeding management. The disorder can be classified into two major categories based on the location of the retained testis: inguinal and abdominal cryptorchidism [1,2,3]. While most cases are unilateral, bilateral cryptorchidism has also been reported and is typically associated with more severe reproductive consequences. The retained testis is usually non-functional in terms of spermatogenesis but will continue to produce androgens, leading to persistent stallion-like behavior [4].

The embryological development of the equine testis begins with the formation of the gonads from the gonadal ridges, arising retroperitoneally caudal to the kidneys around day 40 of gestation [3,5,6]. By day 55, the developing testis is suspended cranially by the cephalic (suspensory) ligament and dorsally by the mesorchium. The interstitial cells begin to proliferate around six weeks of gestation, causing hypertrophy of the testis. At five months, the testis reaches nearly mature stallion size, contacting both the kidney and the deep inguinal ring, and this coincides with elevated maternal estrogen levels [3,6]. Meanwhile, the gubernaculum extends caudally from the testis to the inguinal canal. By day 45, the peritoneum has enveloped the extra-abdominal gubernaculum to form the vaginal process. Around five months, the cephalic ligament regresses, allowing the epididymis to descend into the vaginal process [3].

Later in gestation, between seven and nine months, the gubernaculum shortens and the testis regresses in size, primarily due to loss of interstitial cells. The gubernaculum and epididymis expand in diameter, helping to dilate the vaginal ring and inguinal canal. This, combined with increased intra-abdominal pressure, facilitates passage of the testis into the inguinal canal between 270 and 300 days of gestation. At birth, most testes are within the inguinal canal. The gubernaculum, still prominent, may be mistaken for a testis. Postnatally, the vaginal rings contract within the first few weeks of life and becomes fibrous, which prevents further movement of the testis through the inguinal canal in either direction [2,3,6].

Testicular descent in mammals can be divided into two-phases, influenced by different hormonal signals, mechanical forces, and anatomical structures. The first phase involves the transabdominal migration of the testis, primarily mediated by insulin-like hormone 3 (INSL3), which facilitates gubernacular development. The second, inguinoscrotal phase, is heavily dependent on androgens and the interaction of genitofemoral nerves and other molecular pathways [5,7,8]. Disruptions in this sequence can arrest testicular migration, resulting in retention at various anatomical locations. Histopathological analyses of retained testes in equids have documented immature seminiferous tubules, absence of germ cells, and reduced Sertoli and Leydig cell function [9,10].

The etiology of equine cryptorchidism is multifactorial, with a suggestion of heritability in specific breeds. Previous research has proposed autosomal recessive and polygenic inheritance models, though definitive genetic markers have yet to be identified [11,12]. Given the potential heritability, breeding stallions with a history of cryptorchidism are often excluded from stud programs, although there is variability in how rigorously this policy is applied.

Diagnosis of cryptorchidism has evolved significantly, particularly with the introduction of sensitive hormonal assays. Anti-Müllerian hormone (AMH), produced by Sertoli cells of the testis, serves as a highly specific biomarker for the presence of testicular tissue, including retained testes [9,13,14]. The measurement of basal testosterone levels and testosterone response to human chorionic gonadotropin (hCG) stimulation can also aid in diagnosis, though these methods are less specific and more affected by age, season, and testicular function [9,15]. False negatives can still occur, particularly in prepubertal colts or in cases of testicular degeneration.

Imaging techniques provide additional diagnostic clarity, particularly in cases where hormonal results are equivocal. Ultrasonography, both transabdominal and inguinal, is commonly used to visualize retained testicular tissue, and is especially effective in identifying inguinal retention. Abdominal ultrasound and rectal palpation may also aid in identifying intra-abdominal retention [16,17]. The absence of testicular tissue with ultrasound examination or rectal palpation does not rule out the presence of testicular tissue. Laparoscopy offers both diagnostic and therapeutic utility, allowing direct observation followed by removal of retained testes with minimal invasiveness and shorter recovery times [18,19,20].

The treatment for cryptorchidism is surgical, with the approach determined by the location of the retained testis. Often times, referral to a university or surgical facility is required, which increases time and expense. Inguinal cryptorchidectomy may be performed through an open inguinal incision or using a para-inguinal percutaneous technique. Abdominal cryptorchidectomy typically requires laparoscopy or an open flank approach, with laparoscopy now considered the gold standard for its safety and efficiency [3,18,19]. There are many laparoscopic approaches, all allowing for direct identification and ligation of the spermatic cord with minimal disruption to surrounding tissues. For cases involving incomplete descent or ambiguous anatomy, intraoperative ultrasonography and careful exploration are critical. Selection of the surgical technique often depends on the surgeon’s experience, the facility’s capabilities, and the horse’s temperament and overall health status.

Though neoplastic transformation of retained testes is rare in horses, prolonged retention may predispose them to conditions such as seminomas, teratomas, or Sertoli cell tumors, as has been observed in other species [3,10,21]. As such, early identification and surgical intervention are recommended to reduce the risk of complications and improve long-term outcomes.

The broader implications of cryptorchidism extend to animal welfare, economic considerations, and breeding practices. Unrecognized or untreated cryptorchids may be mistakenly assumed to be geldings, posing behavioral and safety concerns. Additionally, cryptorchid horses may enter breeding programs, potentially propagating the genetic predisposition within certain lines. Therefore, effective diagnostic screening and transparent breeding policies are essential in managing this disorder on a population level [22,23].

Although rare, true monorchidism—the congenital absence of one testis—has been reported in horses but remains difficult to confirm definitively. The condition is often indistinguishable from cases of unilateral castration, colloquially referred to as “hemi-gelding” or hemi-castration, particularly in animals with incomplete medical records or unclear surgical histories [24]. In such cases, hormonal and imaging diagnostics may yield ambiguous results, especially if the retained or absent testis has undergone degeneration or atrophy. Anti-Müllerian hormone levels may be low or undetectable, and ultrasonography may fail to localize testicular tissue, leading to potential misdiagnosis [9,25]. Laparoscopy remains the gold standard for distinguishing between true monorchidism and a retained testis, as direct observation is the only method capable of confirming the complete absence of intra-abdominal or inguinal gonadal tissue. As such, accurate diagnosis in these equivocal cases is critical to avoid misclassification and inappropriate management.

Despite the recognized breed associations, few large-scale studies have evaluated the trends in laterality across different equine populations. The existing literature suggests that larger breed horses have a predisposition for left-sided retention, but ponies have a right-sided predisposition [1,26,27]. These patterns may reflect underlying breed-specific anatomical or genetic factors influencing the testicular descent process.

The present study addresses the lack of studies determining breed predispositions for sidedness by evaluating the distribution of cryptorchid types in Quarter Horses and Thoroughbreds across two major veterinary centers: the Colorado State University Veterinary Teaching Hospital (1984–2014) and Oakridge Equine Hospital (2008–2023). By analyzing breed-specific trends and comparing retention based on sidedness, this study aims to provide a more comprehensive understanding of the epidemiological and clinical characteristics of equine cryptorchidism.

Understanding these patterns is crucial for improving diagnostic algorithms, surgical planning, and prevention strategies through informed breeding decisions. With advances in hormonal testing, imaging modalities, and minimally invasive surgery, clinicians are better equipped to diagnose and manage cryptorchidism. However, ongoing research is needed to elucidate the genetic and developmental underpinnings of this condition and to enhance the accuracy, efficiency, and accessibility of diagnostic protocols in equine practice.

## 2. Materials and Methods

Medical records from the Colorado State University Veterinary Teaching Hospital (CSU VTH) spanning 1984 to 2014 and from Oakridge Equine Hospital (OEH) between 2008 and 2023 were retrospectively reviewed to identify horses diagnosed with cryptorchidism. Data collection focused on horses that underwent evaluation for cryptorchid castration, and only cases with clearly documented laterality (left, right, or bilateral) were included in the analysis. A total of 777 horses met criteria and were included in the analysis.

Information extracted from surgical reports and medical records included the horse’s age, breed, and the laterality of testicular retention. For the purpose of this study, a cryptorchid horse was defined as any intact male in which one or both testes had failed to descend into the scrotum by the time of presentation for castration. Testes were categorized by laterality, but not by specific anatomical location (inguinal vs. abdominal) due to inconsistencies in record keeping and surgical descriptions. When it was not possible to clearly determine the anatomic site of retention, that data was excluded from the study.

Cases were excluded if data on laterality was missing, ambiguous, or if the medical record did not include a definitive diagnosis of cryptorchidism. Horses in which only one testis was identified during surgery, with insufficient documentation to confirm whether the condition was due to true monorchidism or prior unilateral castration, were also excluded. True monorchidism, defined as a congenital absence of one testis, is considered extremely rare in horses and cannot be definitively diagnosed without histopathologic and developmental evidence. In practice, it is often indistinguishable from incomplete castration in horses with limited or missing historical records. Therefore, in order to maintain the integrity of the dataset and ensure consistency in diagnosis, suspected monorchid cases were not included in the final analysis.

### Statistical Analysis

All data were copied into Excel and converted to .csv files for import into R for analysis [28,29]. Initial counts were converted to contingency tables and analyzed for the presence of any differences between groups utilizing graphical representations and both chi-square and Fisher’s exact tests via the base ‘*stats*’ package. As the *p*-values were significant (indicating some differences between groups), a multinomial model (package ‘*nnet*’ for analyzing count data) was used to evaluate differences between the breeds in laterality of cryptorchidsm [29]. Specific comparisons between individual breeds were then evaluated based on the multinomial model via differences in estimated marginal means (package ‘*emmeans*’) using a treatment-vs-control strategy comparing Quarter horse to other breeds [30]. As there were no differences in groups when comparing instance of bilateral cryptorchidism, this term was dropped from the model, and the final modeling was performed comparing only right to left cryptorchidism. All analysis was performed using R version 4.5.0 (‘How About a Twenty-Six’) and packages as listed above assuming a *p*-value of 0.05 as statistically significant. Code and data used for this analysis are available at https://github.com/gregg-g/Fain_Hend_crypt_23.git (accessed on 19 March 2024).

## 3. Results

A total of 777 horses met the inclusion criteria, including 565 Quarter Horses, 127 Thoroughbreds, 19 Arabians, 19 mixed breed, 15 Warmbloods, 14 ponies, 12 Saddlebreds, and 6 Draft Horses. Quarter Horses were overrepresented in the dataset, which likely reflects the patient population at CSU VTH and OEH rather than a true breed predisposition.

**Figure 1:**
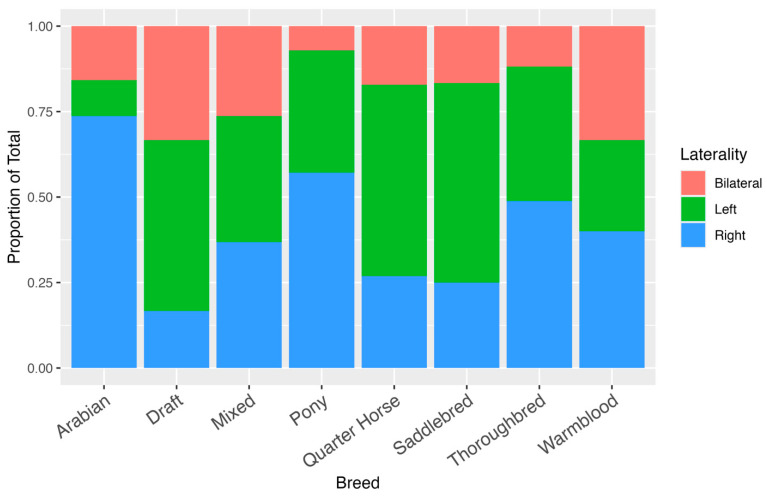
Proportion of cryptorchid horses by laterality (bilateral, left, or right) within each breed.

**Figure 2:**
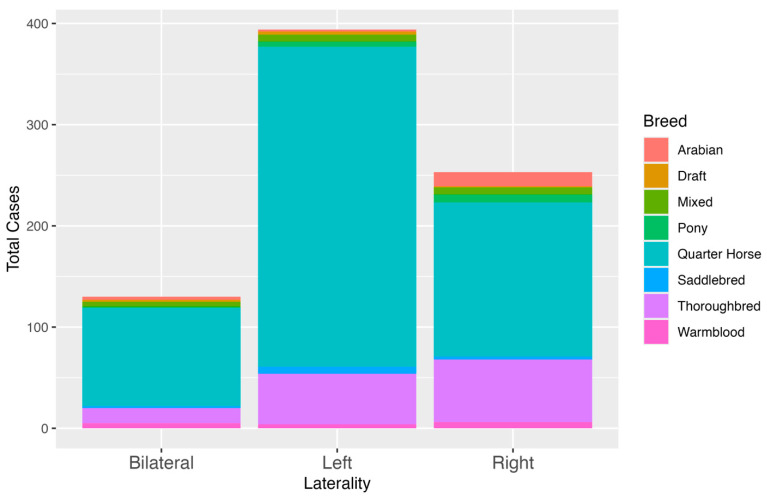
Total number of cryptorchid horses by laterality and breed.

Among the 565 Quarter Horses included in the study, 316 (55.9%) presented with left-sided cryptorchidism, 152 (26.9%) with right-sided cryptorchidism, and 97 (17.2%) with bilateral retention. Table 1. When bilateral cases were factored into the laterality distribution, the left testis was retained in a total of 413/565 (73.1%) of Quarter Horse cases. This substantial predominance of left-sided retention supports prior anecdotal and published observations indicating that left-sided cryptorchidism is more commonly encountered in this breed. Figure 1 and Figure 2

Of the 127 Thoroughbreds, 50 (39.4%) had a left-retained testicle, 62 (48.8%) had a right-retained testicle, and 15 (11.8%) presented with bilateral cryptorchidism. When bilateral cases were distributed across both sides, the right testicle was retained in 77/127 (60.6%) Thoroughbreds, revealing a significant right-sided bias in this breed (*p* = 0.0144).

Arabians, though a small portion of the study population, demonstrated a clear right-sided predominance. Of the 19 Arabian horses evaluated, 2 (10.5%) had a left-retained testicle, 14 (73.7%) had a right-retained testicle, and 3 (15.8%) were bilaterally cryptorchid. When including bilateral cases, the right testicle was retained in 17/19 (89.5%) of Arabian cases, suggesting a potentially strong right-sided predisposition in this breed.

Quarter Horses were significantly more likely to present with left-sided testicular retention than Thoroughbreds (OR, 0.388; 95% CI, 0.255–0.590) and Arabians (OR, 0.069; 95% CI, 0.0154–0.306). Ponies, Warmbloods, Saddlebreds, mixed breeds, and Draft Horses were represented in lower numbers, limiting the ability to draw statistically meaningful conclusions about breed-specific laterality trends within these groups. While ponies appeared to exhibit a trend toward right-sided retention, no statistical conclusion can be drawn due to the small sample size. There were no notable differences in the incidence of bilateral cryptorchidism between breeds.

## 4. Discussion

This study analyzed cryptorchidism in Quarter Horses and Thoroughbreds over a nearly four-decade period, revealing clear tendencies in laterality and breed predispositions. Our findings align with historical reports that cryptorchidism is more commonly left-sided in Quarter Horses, while Thoroughbreds show a higher incidence of right-sided retention [1,26,27]. These results suggest a possible genetic influence on testicular descent, consistent with reports of potential heritability in other breeds [31].

Despite some well-documented breed associations, few large-scale studies have systematically evaluated laterality and anatomical presentation of cryptorchidism across different equine populations. While earlier studies suggested no significant difference in left versus right testicular retention overall [1,26], these analyses did not stratify by breed. This may have masked breed-specific tendencies due to opposing laterality patterns. For example, ponies appear predisposed to right-sided retention, whereas larger breeds may more frequently retain the left testis [32]. In our study population of Quarter Horses, the left testis was significantly more likely to be retained. Hormone analysis is beneficial in determining the presence of testicular tissue [33,34,35].

Surgically, the approach depends on the testis location. Laparoscopic techniques have become the gold standard for abdominal cryptorchids due to their minimally invasive nature and reduced complication rates [18,19,20]. Laparoscopy provides the opportunity to directly evaluate the mesorchium and the inguinal canals, minimizing the potential of leaving testicular tissue behind. Standing laparoscopy also provides the surgeon the opportunity to remove testes from either side of the abdomen from a single approach in many cases [18]. However, the authors recommend preparing both flanks when pursuing a standing laparoscopic approach in the case of a horse with unknown castration history. Inguinal retention can also be addressed via open para-inguinal or open direct inguinal approaches, with success rates exceeding 90% [3,36].

While cryptorchidism is not typically life-threatening, it carries significant economic implications. Colts that might otherwise be viable breeding prospects are often excluded from the gene pool, diminishing their market value and eliminating potential revenue from stud fees. Additionally, surgical correction of cryptorchidism is generally more complex and costly than routine field castration. This is especially true when a horse has had the descended testis removed, records are lost or ambiguous, and laparoscopic surgery is not an option due to availability or financial constraints.

Breed-specific predispositions observed in this study suggest a role for selective breeding strategies in reducing the incidence of cryptorchidism. Further genetic studies are warranted, especially as cryptorchidism can impact stallion fertility and long-term reproductive performance [11,24,37]. Additionally, developmental anatomy studies offer insight into normal and aberrant testicular descent across species [6,38,39,40].

Future work should include molecular profiling and prospective evaluation of diagnostic tools to further improve the accuracy and cost-effectiveness of cryptorchid diagnosis and treatment in equine practice [25]. Additional work should also include the anatomic localization of cryptorchid testicles in inguinal or abdominal locations. There is also anecdotal evidence that would point away from cryptorchidism being a heritable trait. It would be valuable to perform a multi-institution project to assess the actual heritability characteristics of cryptorchidism. This report should be helpful in directing the veterinarian as to the most effective surgical approach for each of the horse breeds represented in this study. This is especially true in the case of unknown castration history or when a horse has had one testis removed but the records are incomplete in identifying which testis was removed.

## 5. Conclusions

Cryptorchidism is a clinically significant congenital condition in stallions, with implications for behavior, reproductive management, and surgical intervention. This retrospective study analyzed trends in anatomical presentation and laterality among cases seen at two major equine veterinary hospitals. Quarter Horses are overrepresented in the study population as this is the predominant breed seen at both CSU and OEH. Quarter Horses are more likely to be left-sided cryptorchid, while Thoroughbreds, Arabians, and likely ponies as well are more likely to be right-sided cryptorchid. There was no breed predisposition identified with bilateral cryptorchidism.

Overall, this study highlights a previously underreported left-sided predisposition in Quarter Horses with cryptorchidism. Recognition of this tendency may improve preoperative planning, allowing veterinarians to target the most probable location of a retained testis and thereby reduce anesthetic time and surgical morbidity. Our findings support the hypothesis that Quarter Horses are more likely to have a left-retained testis than a right-sided or bilateral retention. These results provide clinically relevant insight for managing cryptorchid horses, particularly in cases with unknown castration status, and underscore the need for breed-specific approaches in both diagnosis and surgical treatment.

Future research should aim to clarify the molecular mechanisms of testicular descent, evaluate long-term surgical outcomes, and further refine diagnostic protocols to support clinicians in delivering efficient and effective care for cryptorchid horses.

## Figures and Tables

**Table 1:** Classification of cryptorchid sidedness by breed.

Breed	Bilateral	Left	Right
Arabian (ARAB)	3 (15.8%)	2 (10.5%)	14 (73.7%)
Draft (DR)	2 (33.3%)	3 (50%)	1 (16.7%)
Mixed Breed (MX)	5 (26.3%)	7 (36.8%)	7 (36.8%)
Pony	1 (7.1%)	5 (35.7%)	8 (57.1%)
Quarter Horse (QH)	97 (17.2%)	316 (55.9%)	152 (26.9%)
Saddlebred (SAD DLE)	2 (16.7%)	7 (58.3%)	3 (25.0%)
Thoroughbred (TB)	15 (17.2%)	50 (39.4%)	62 (48.8%)
Warmblood (WB)	5 (33.3%)	4 (26.7%)	6 (40.0%)

## Data Availability

Code and data used for this analysis are available at https://github.com/gregg-g/Fain_Hend_crypt_23.git. URL accessed on 19 March 2024.

## Abbreviations

The following abbreviations are used in this manuscript:

ARAB	Arabian Horse
DR	Draft
MX	Mixed Breed
PONY	Pony
QH	Quarter Horse
SADDLE	Saddlebred
TB	Thoroughbred
WB	Warmblood
